# How does robotic surgery affect gynecology patient care?

**DOI:** 10.1007/s11701-024-01955-1

**Published:** 2024-06-19

**Authors:** Sibel Arslan, Katri Vehviläinen-Julkunen, Anndra Parviainen

**Affiliations:** 1 Department of Surgical Diseases Nursing Science, Faculty of Health Sciences, Kilis 7 Aralık Universty, Kilis, Türkiye; 2 Department of Nursing Science, Faculty of Health Sciences, Kuopio University Hospital, University of Eastern Finland, Kuopio, Finland; 3 Department of Nursing Science, Faculty of Health Sciences, School of Medicine, Institute of Public Health and Clinical Nutrition, Universty Eastern of Finland, Kuopio, Finland

**Keywords:** Robotic surgery, Patient need, Robotic surgery nursing, Gynecological robotic surgery

## Abstract

The aim of this review is to map the current research on the needs of gynecological patients treated with robotic surgery. Systematic Rapid Review. Pubmed, Web of Science, Google Scholar. Search was limited from the years 2017–2021. The Preferred Reporting Items for Systematic Reviews and Meta-Analysis (PRISMA) statement was followed. Rapid review is a synthesis of information produced in a shorter time than systematic reviews, which allows clinical nurses to access evidence in the decision-making process. The methodological steps implemented were the following: (1) needs assessment and topic selection, (2) study development, (3) literature search, (4) screening and study selection, (5) data extraction, (6) risk-of-bias assessment and (7) knowledge synthesis. The search yielded 815 articles, 746 were excluded after screening the title and abstract, and 69 full-text syntheses were performed. Only 10 articles were included in the final analysis. This research evaluated the effects of robotic surgery on the patient under seven themes; operative time, length of stay, complications, estimated blood loss, pain, survivor, and conversion. Five studies were on endometrial cancer, one study on gynecologic cancer, two studies on hysterectomy, one study on patient safety, and one study on cervical cancer. The results show that robotic surgery can change the needs of patients by solving ongoing problems in gynecological patients. This requires a better understanding of robotic surgery procedures while facilitating nursing care over patient care.

## Introduction

Robotic surgery influences nursing care and have an impact on patients’ needs. The main role of nursing in the perioperative period is to ensure patient safety [1]. Robotic surgery is one of the latest surgical innovations in many countries worldwide [2] Robotic surgical systems are used in various procedures such as cardiothoracic surgery, urology, endocrine surgery, metabolic and bariatric surgery, head and neck surgery and all the intra-abdominal surgeries.

Using a computer-assisted platform, robotic surgery is a technologically more sophisticated extension of traditional laparoscopy [2]. Nurses working with robotic systems face a number of conceptual and technical challenges. Important behavioral markers namely eye contact and anticipatory movements are compromised in the operating theater. In addition, robotic surgery demands high technical competence and a more active role from the operating room nurse, whose responsibilities are to assist the surgeon, to pay attention to the rules of asepsis by distinguishing the sterile and non-sterile parts of the robot, to place the robot arms [3].

The trends of using robotic surgery by year are given in Table 1 [4, 5]. Robotic surgery has several advantages compared to the previous; ergonomically superior, tremor-control, camera stabilization, depth perception due to the 3D camera (less blood loss, reduced transfusions, reduced complications), and lower conversion rate to open surgery [2, 6]. Even so advantages of laparoscopic surgery over robotic surgery haptic feedback, cost-effective, and flexibility instrument configuration [6]. Da Vinci systems include EndoWrist instruments that provide greater feedback to the surgeon. Although these arms increase mobility through combinations of placement, tilt, yaw, roll, and grip, they provide limited tactile feedback [7, 8].

**Table 1:** Trends of using robotic surgery

Year	Name	Usage areas
1985	PUMA 200 (first robot surgeon)	Neurosurgical biopsies
1988	UROBOT	Urologic
1990	“Master–slave system” identified	Robotic based surgery
1993	Automated endoscopic system for optimal positioning (AESOP)	Laparoscopic cholecystectomy, hernioplasty, fundoplication, colectomy
1998	ZEUS robotic surgical system	Urologic, gynecologic, heart surgeries
2000	Da Vinci ® FDA approved	Abdominal surgeries and commonly urologic, gynecological surgeries, including prostatectomy for cancer and hysterectomy for benign diseases

Despite the innovational advantages of robotic surgery that it can bring, there are studies that argue that there is no clinically significant difference between robotic surgery and other surgical methods [2, 9, 10]. Compared to other surgical methods, patients who underwent robotic-assisted surgery may need different patient care due to shorter operating times and shorter hospital stays [11]. Hence, this rapid review was conducted based on the different patient care results that robotic surgery brings. In this rapid review, the focus is on the needs of gynecology patients where robotic surgery is used the most and how it can affect nursing care.

## Aim

The aim of this review is to map the current research on the needs of gynecological patients treated with robotic surgery.

## Methods

### Design

Rapid review is a synthesis of information produced in a shorter time than systematic reviews, which allows clinical nurses to access evidence in the decision-making process [12–14]. In line with the study design and aims, the quality appraisal of the studies was not performed while a selective process of data extraction was applied. The methodological steps implemented were the following: (1) needs assessment and topic selection, (2) study development, (3) literature search, (4) screening and study selection, (5) data extraction, (6) risk-of-bias assessment and (7) knowledge synthesis [15, 16].

### Needs assessment, topic selection and study development

The rapid use of robotic surgery in the field of gynecology and how this changes patient needs and nursing care were deemed necessary by literature analysis [1, 6]. The needs we mention here arise from the changing results with robotic surgery. For example, does the change in surgery duration create new needs for patients or how is nursing care affected by this situation?

### Screening and study selection

The following databases were utilized: Pubmed, Web of Science, and Google Scholar. Search was limited from the years 2017–2021. Data were collected using the following keywords; robotic surgery, patient need and robotic surgery nursing. The search yielded 815 articles, 746 were excluded after screening the title and abstract screened. 69 full-text syntheses were performed. Among 69 studies, 15 studies were included because they were related to gynecology. Of the 54 studies, 5 were excluded because they were head and jaw, 11 were urology, 21 were general surgery, 4 were thoracotomy, 8 were transoral, 2 were nephrectomy, and 3 were perioperative studies. Five of the 15 gynecology studies were excluded because the methods were different in specific patient groups. Only 10 articles were included in the final analysis (Table 2).

**Table 2:** Characteristics of the articles (*n* = 10)

Authors, name and countries	Study desingns	Study focus	What type of desease/illness?
Lindfors et al. [17], Sweden (2020)	An observational cohort study	Patients *n* = 217	Endometrial cancer
Planque et al. [18], France (2018)	Retrospective Study	Patients *n* = 77	Endometrial cancer
Gallotta et al. [19], Italy (2018)	Retrospective Cohort Study	Elderly and very elderly patients *n* = 204	Gynecologic cancer
Herrinton et al. [20], California (2019)	Retrospective Cohort Study	Patients *n* = 7345	Hysterectomy Benign conditions
Balafoutas et al. [21], Germany (2020)	Retrospective Analysis	Procedures *n* = 110	Patient safety
Sofer et al. [22], Israel (2020)	Retrospective Study	Obese women *n* = 138	Endometrial cancer
Silva e Silva et al. [23], Brazil (2018)	Prospective Randomized Study	Patients *n* = 89	Endometrial cancer
Sinha et al. [24], India (2019)	Retrospective review	Patients *n* = 165	Hysterectomy
Liu et al. [25], Taiwan (2019)	Retrospective Study	Patients *n* = 39	Cervical cancer
Soto et al. [26], Massachusetts (2017)	Randomized Control Trial	Patients *n* = 73	Endometriosis

**Fig. 1:**
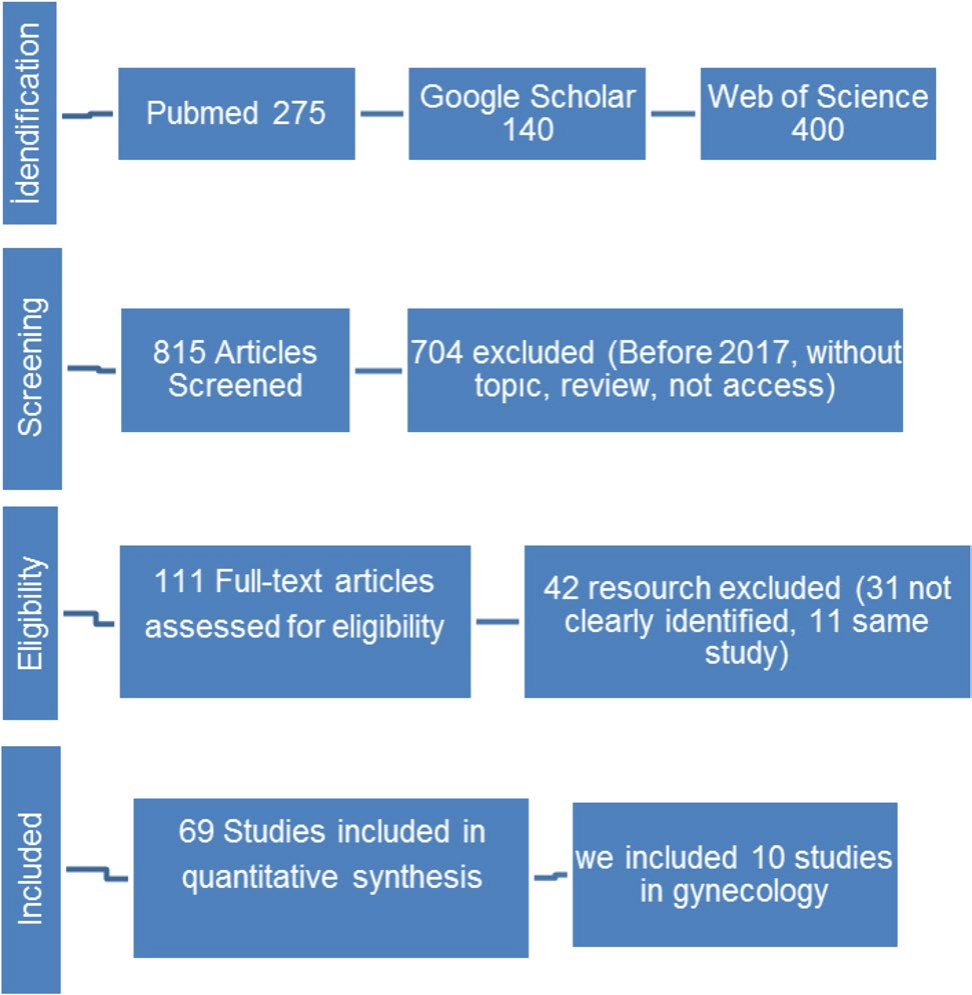
Flowchart for the search and study selection process following the PRISMA guidelines [27]

One researcher (SA) performed the literature search and two researchers (SA and KVJ) worked independently to evaluate study eligibility based on the title and abstract screening of each study that emerged. All articles considered to be eligible were then retrieved in full-text format. Two researchers (SA and KVJ) independently read the full text of all articles and evaluated their inclusion. The inclusion of any article was decided upon a joint agreement. The full process of study inclusion (from the database search to the inclusion phase) is explained in Fig. 1.

### Data analysis

Data were analyzed using Braun and Clarke’s thematic analysis method [28]. Theme topics were found by two independent researchers (SA, KVJ) using the thematic analysis method (Table 3). These themes; operative time, length of stay, complications, estimated blood loss, pain, survivors and conversion.

**Table 3:** Data extraction table (*n* = 10)

Author and year	Operative time	Length of stay hospital	Complications	Estimated blood loss	Pain	Survivor	Conversion
A. Lindfors et al. [17], (2020) Robotic or open surgery in obese women with endometrial cancer	Robotic surgery shorter operation time	Robotic surgery shorter postoperative stay (*P* < 0.001)	Obese patients in terms of a reduced complication rate. there was a significant reduction in complications in the robotic group	Robotic surgery reduction in estimated blood loss	–	Robotic surgery not found a significant independent factor for survival. (*P* = 0.024)	–
H. Planque et al. [18], Comparison of robotic surgery in obese and non-obese patients endometrial cancer	There was no difference for the total operative time (*P* = 0.50)	The median hospital stay was 3 days (*P* = 0.92)	There was no statistical difference for postoperative complications (*P* = 1)	–	–	–	One patient (not obese) had a laparoconversion
Gallotta et al. [19], Robotic Surgery in Elderly and Very Elderly Gynecologic Cancer Patients	The Elderly and Very elderly were comparable in terms of operative time. (*P* = 0.311)	The median length of hospital stay was 2 days in each group. (*P* = 0.172)	A total of 11 patients (5.6%) had early postoperative complications	The Elderly and Very elderly were comparable in terms of blood loss. (*P* = 0.919)	–	–	A total of 7 (3.4%) conversions to open surgery were registered (*P* = 1.00)
Herrinton et al. [20],	The average operative time was reduced (*P* < 0.0001)	mean length of stay was similar for the robotic and conventional group	Among noncomplex cases, robotic surgery was associated with complications of the urinary tract (*P* = 0.04). No other significant differences in risk of complications between conventional and robotic cases	Robotic surgery reduction in estimated blood loss	–	–	–
Balafoutas et al. [21],	–	–	Ninety (81.8%) procedures were completed without any problems	No blood transfusion was needed	–	–	No need for conversion to laparotomy was observed
Sofer et al. [22],	Robotic surgery was longer operating theater time (*P* < 0.001)	Robotic surgery was associated with shorter hospital stays (*P* < 0.0001)	Robotic surgery fewer postoperative complications (*P* = 0.0008)	–	–	5-year survival was higher in the robotic surgery group. Quality of life measures were better after robotic surgery	–
Silva e Silva A et al. [23],	In our study, robotic surgery was more time consuming than traditional laparoscopic surgery	The median hospital stay was was similar in both groups	Eight major complications were registered in each group	Estimated blood loss was higher in the robotic surgery group, but not significant (*P* = 0.64)			One patient in the robotic surgery group was converted to laparotomy
Sinha R et al. [24], Comparison of Robotic and Laparoscopic Hysterectomy for the Large Uterus	Robotic surgery took longer operating time (*P* = 0.006)	Postoperative stay was similar in both groups (1.4 days)		Robotic surgery had less drop in Hb (*P* < 0.001)	Requirement of intravenous analgesia was significantly lower in the Robotic surgery group (*P* < 0.001)		Open conversion rate was 4.3% (RH) and 10.9% (LH) but not significant
Liu et al. [25], Comparative Analysis between the Robotic and Abdominal Approaches	Robotic surgery shorter operation time *P* < 0.001	Hospital stay (days) reduced robotic surgery *P* < 0.001	had in 2 patients in robotic surgery intraoperative period *P* = 0.49 had in 3 patients in robotic surgery postoperative complications *P* = 0.814	Robotic surgery reduction in estimated blood loss *P* < 0.001	Postoperative pain score (VAS) and 24-h postoperative pain score (VAS) less in robotic surgery *P* < 0.001	–	No conversion to laparotomy was needed in the robotic surgery group
Soto et al. [26], Laparoscopy vs. Robotic Surgery for Endometriosis (LAROSE): a multicenter, randomized, controlled trial	There were no statistical differences in operative time	–	There were no differences in postoperative complications	There were no statistical differences in blood loss	–	–	There were no differences in rates of conversion to laparotomy in the two arms

### Risk-of-bias assessment and knowledge synthesis

Several strategies were used to avoid bias: (a) the review team shared each step of the study inclusion and exclusion process; (b) the three main databases and reference lists of the included studies were carefully reviewed; (c) data extraction was performed by two reviewers; and (d) articles in English are included.

Thematic analysis was conducted by systematically coding and categorizing the textual information obtained from the included articles of how robotic surgical treatment will change patient outcomes.

## Results

Table 3 presents the characteristics of the included ten studies. Papers were published within the year 2017–2021 involving gynecologic participants. Five studies were on endometrial cancer, one study on gynecologic cancer, two studies on hysterectomy, one study on patient safety, one study on cervical cancer. The country of origins were from Sweden, France, Italy, California, Germany, Israel, India, Taiwan, Brazil, and Massachusetts.

### Reported content of robotic surgeries

Operative time = In 3 studies, it was stated that robotic surgery shortened the operation time, in 3 studies it did not affect the operation time, and in 3 studies it extended the operation time.

Length of stay = It was seen that robotic surgery did not affect the length of stay in hospital in 5 studies and robotic surgery treatment in 3 studies significantly reduced the length of stay in hospital.

Complications = In the studies, it was determined that postoperative and intraoperative complications can be seen in robotic surgery, that complications decreased in some studies, and that it was not significant between the groups in some comparative studies.

Estimated Blood Loss = Most of the studies show that the estimated blood loss is reduced in the treatment of robotic surgery.

Pain = In two studies, it was stated that robotic surgery treatment significantly reduced pain.

Survior = Studies have shown no significant outcome of robotic surgery on survival. Only the results of two studies were looked at.

Conversion = It was stated in studies that there was a transition from robotic surgery to open surgery.

## Discussion

How the results might affect nursing care and patient needs were interpreted in line with the literature. Changes in the themes found may require new changing needs. Shorter stay for patients, shorter or longer operation time, less pain etc. The effects of robotic surgery on the patient were evaluated under seven themes.

### Operative time

The operative time of robotic surgery was evaluated in the articles. There are different results that show the operating time of robotic surgery to be shorter [17, 20, 25] and longer [22–24] than other methods. While there are studies showing that the duration of robotic surgery is longer in endometrial cancer surgeries [22, 23] one study [17] has shown it to be shorter. When the robotic surgery operative time was compared in hysterectomy and abdominal approaches, it was observed that there were different results [20, 24, 25]. No significant difference was found in studies comparing the duration of robotic surgery in obese and elderly patients [18, 19]. It has been stated that the surgeon’s experience and the training of the operating room staff will contribute to shortening the operating time [7, 29, 30]. In terms of nursing care, job descriptions in robotic surgery can be made and situations that prolong the operation time can be determined.

### Length of stay

Studies have shown that robotic surgery does not prolong hospital stay. However, in five studies out of ten no effect of robotic surgery on length of stay was observed, while in three studies it was observed that it shortened the length of stay [17, 22, 25].

### Complications, estimated blood loss, pain

While there are results showing a decrease in complications in studies, there are also results that do not find a significant difference. It cannot be said that robotic surgery treatment completely reduces complications after gynecological surgery. Compared to laparoscopic surgery, robotic surgery has been shown to reduce estimated blood loss. Only one study showed greater blood loss in robotic surgery. In two studies comparing robotic surgery and laparoscopic surgery, pain was found to be significantly reduced in patients after robotic surgery. Reducing pain after surgery will also reduce patients’ need for painkillers. This may facilitate nursing care.

### Survivor, conversion

The effect of robotic surgery on survival was examined in two studies. In one study, robotic surgery was not found to be significant in survival, while in another it was found to be more effective in five-year survival. Conversion was evaluated in six studies. Conversion was not required in two of the studies [21, 25]. In a study comparing robotic surgery and laparoscopic treatments, the conversion rate of robotic surgery to open surgery was found to be lower than laparoscopy [24]. Transitions from robotic surgery to laparotomy were observed in two studies [18, 23]. In a study evaluating the treatment of elderly and very elderly patients with robotic surgery, a total of seven patients were converted to open surgery [19]. Conversion is a new definition developed by minimally invasive surgery for nurses. The transformation from robotic surgery to laparotomy or open surgery is a new role for nursing.

### Effectiveness of robotic surgery for gynecological patients

RAS (robot-assisted surgery) is a system in which robots are used in a minimally invasive surgery method. It functions under the guidance of robot surgeons [31]. It is argued that with an experienced and trained team, the effectiveness of robotic surgery will increase and eliminate complexity in difficult parts of the body [29, 31]. There are studies showing that robot-assisted surgery is safe and effective in gynecological patients [29, 32, 33]. According to the patient results in our review, it appears that robotic surgery can also be used in gynecological patients. However, when we compare the results with laparoscopic surgery, we cannot always say that robotic surgery is superior. This may vary depending on the type of surgery and patient characteristics (age, obesity).

### Nursing care needs for gynecologic patients who underwent robotic surgery

The surgical center is a risky area of the hospital where critical decisions are made, where emergency or elective surgical diagnoses and treatments are made. Patient safety and comfort are considered the primary areas in robotic surgery. Organizing the perioperative period and continuity of care is the responsibility of the nurse. Robotic surgery has increased with technological advances, does not change the reality of care, which is the basic duty of the nurse. However, these practices may add different roles to nurses. In addition, nurses also play a role in robot management [1, 34].

The themes of our research, operation time, conversion and estimated blood loss, are the results that affect the intraoperative period. In our rapid review there are studies showing that robotic surgery prolongs the operation time and concluding that conversion to laparoscopy or open surgery has occurred. We conclude that the estimated blood loss is reduced more with robotic surgery. We believe that there is a need for nursing research intraoperative gynecological patients using robotic surgery.

Prolonged hospital stay, complications and pain are undesirable situations in the postoperative period. The solution of robotic surgery in these areas can increase the quality of nursing care and patient satisfaction.

### Limitations

Rapid reviews are syntheses in which systematic reviews are simplified. However, it has been stated that it is useful for nurses in clinical practice [12]. Although the search, screening and abstraction stages were not carried out as meticulously as a systematic review, this study provides up-to-date information to nurses on the subject.

A further limitation was introduced by simplifying the search and screening steps, creating the risk of missing some relevant evidence. However, the initial decision-making process of the study and the inclusion of the articles were decided by two researchers in a face-to-face meeting. Additionally, the researchers’ expertise in the fields of nursing, surgical nursing, and gynecology nursing was effective in reducing the risk of bias.

## Conclusion

The results show that robotic surgery can change the needs of patients by solving ongoing problems in gynecological patients. This requires a better understanding of robotic surgery procedures while facilitating nursing care over patient care.

## Data Availability

No datasets were generated or analysed during the current study.

## References

[1] Martins RC, Trevilato DD, Jost MT, Caregnato RCA (2019) Nursing performance in robotic surgeries: integrative review. Rev Bras Enferm 72(3):795–800. 10.1590/0034-7167-2018-0426 31269148 10.1590/0034-7167-2018-0426

[2] Varghese A, Doglioli M, Fader AN (2019) updates and controversies of robotic-assisted surgery in gynecologic surgery. Clin Obstet Gynecol 62(4):733–748. 10.1097/GRF.0000000000000489 31524659 10.1097/GRF.0000000000000489 PMC6944326

[3] Vigo F, Egg R, Schoetzau A, Montavon C, Brezak M, Heinzelmann-Schwarz V, Kavvadias T (2022) An interdisciplinary team-training protocol for robotic gynecologic surgery improves operating time and costs: analysis of a 4-year experience in a university hospital setting. J Robot Surg 16:89–96. 10.1007/s11701-021-01209-4 33606159 10.1007/s11701-021-01209-4 PMC8863701

[4] Ghezzi TL, Corleta OC (2016) 30 Years of robotic surgery. World J Surg 40:2550–2557. 10.1007/s00268-016-3543-9 27177648 10.1007/s00268-016-3543-9

[5] Saini S, Orlando MF, Pathak PM (2022) Intelligent control of a master-slave based robotic surgical system. J Intell Rob Syst 105(4):2–20. 10.1007/s10846-022-01684-3

[6] Clair KH, Tewari KS (2020) Robotic surgery for gynecologic cancers: indications, techniques and controversies. J Obstet Gynaecol Res 46(6):828–843. 10.1111/jog.14228 32410262 10.1111/jog.14228 PMC7387116

[7] Beste TM, Nelson KH, Daucher JA (2005) Total laparoscopic hysterectomy utilizing a robotic surgical system. J Soc Laparoendosc Surg 9(1):13–15 PMC3015554 15791963

[8] Williamson T, Song SE (2022) Robotic surgery techniques to improve traditional laparoscopy. J Soc Laparoendosc Surg 26(2):e2022.00002. 10.4293/JSLS.2022.00002 10.4293/JSLS.2022.00002 PMC9135605 35655469

[9] Rosero EB, Kho KA, Joshi GP (2013) Comparison of robotic and laparoscopic hysterectomy for benign gynecologic disease. Obstet Gynecol 122:778–786. 10.1097/AOG.0b013e3182a4ee4d 24084534 10.1097/AOG.0b013e3182a4ee4d PMC4361072

[10] Lim PC, Crane JT, English EJ, Farnam RW, Garza DM, Winter ML, Rozeboom JL (2016) Multicenter analysis comparing robotic, open, laparoscopic, and vaginal hysterectomies performed by high-volume surgeons for benign indications. Int J Gynecol Obstet 133:359–364. 10.1016/j.ijgo.2015.11.010 10.1016/j.ijgo.2015.11.010 26952352

[11] Long E, Kew F (2018) Patient satisfaction with robotic surgery. J Robot Surg 12:493–499. 10.1007/s11701-017-0772-3 29288373 10.1007/s11701-017-0772-3

[12] O’leary DF, Casey M, O’Connor L, Stokes D, Fealy GM, O’Brien D, Smith R, McNamara M, Egan C (2017) Using rapid reviews: an example from a study conducted to inform policy making. J Adv Nurs 73(3):742–752. 10.1111/jan.13231 10.1111/jan.13231 27943377

[13] Tricco AC, Antony J, Zarin W, Strifler L, Ghassemi M, Ivory J, Perrier L, Hutton B, Moher D, Straus SE (2015) A scoping review of rapid review methods. BMC Med 13(224):2–15. 10.1186/s12916-015-0465-6 26377409 10.1186/s12916-015-0465-6 PMC4574114

[14] Seibert K, Domhoff D, Bruch D, Schulte-Althoff M, Fürstenau D, Biessmann F, Wolf-Ostermann K (2020) A rapid review on application scenarios for artificial intelligence in nursing care. J Med Internet Res:26522. 10.2196/preprints.26522 10.2196/26522 PMC8669587 34847057

[15] Langlois EV, Straus SE, Antony J, King VJ, Tricco AC (2019) Using rapid reviews to strengthen health policy and systems and progress towards universal health coverage. BMJ Glob Health 4(1):e001178. 10.1136/bmjgh-2018-001178 30899562 10.1136/bmjgh-2018-001178 PMC6407563

[16] Mansutti I, Achil I, Gastaldo CR, Pires CT, Palese A (2022) Individuals with hearing impairment/deafness during the Covid-19 pandemic: a rapid review on communication challenges and strategies. J Clin Nurs 32:4454–4472. 10.1111/jocn.16572 36320127 10.1111/jocn.16572

[17] Lindfors A, Heshar H, Adok C, Sundfeldt K, Dahm-Kähler P (2020) Long-term survival in obese patients after robotic or open surgery for endometrial cancer. Gynecol Oncol 158:673–680. 10.1016/j.ygyno.2020.05.684 32527569 10.1016/j.ygyno.2020.05.684

[18] Planque H, Martin-Françoise S, Lequesne J, Le Brun JF (2018) Robotic surgery in endometrial cancer: feasibility in obese patients. Gynecol Obstet Fertil Senol 46:625–631. 10.1016/j.gofs.2018.07.002 30115552 10.1016/j.gofs.2018.07.002

[19] Gallotta V, Conte C, D’Indinosante D, Federico A, Biscione A, Vizzielli G, Bottoni C, Carbone MV, Legge F, Uccella S, Ciocchetti C, Russo A, Polidori L, Scambia G, Ferrandina G (2018) Robotic surgery in elderly and very elderly gynecologic cancer patients. J Minim Invasive Gynecol 25(5):872–877. 10.1016/j.jmig.2018.01.007 29339300 10.1016/j.jmig.2018.01.007

[20] Herrinton LJ, Raine-Bennett T, Liu L, Alexeeff SE, Ramos W, Suh-Burgmann B (2019) Outcomes of robotic hysterectomy for treatment of benign conditions: influence of patient complexity. Perm J 24(2020). 10.7812/TPP/19.035 10.7812/TPP/19.035 PMC6972554 31905335

[21] Balafoutas D, Wöckel A, Wulff C, Joukhadar R (2020) Implementation of robotic gynecological surgery in a German University Hospital: patient safety after 110 procedures. Arch Gynecol Obstet 302(2020):1381–1388. 10.1007/s00404-020-05751-8 32844240 10.1007/s00404-020-05751-8 PMC7584536

[22] Sofer A, Magnezi R, Eitan R, Raban O, Tal O, Smorgic N, Vaknin Z (2020) Robotic vs. open surgery in obese women with low-grade endometrial cancer: comparison of costs and quality of life measures. Israel J Health Policy Res 9(60):2–8. 10.1186/s13584-020-00412-2 10.1186/s13584-020-00412-2 PMC7607708 33138857

[23] Silva e Silva A, Carvalho JP, Anton C, Fernandes R P, Baracat EC, Carvalho JP (2018) Introduction of robotic surgery for endometrial cancer into a Brazilian cancer service: a randomized trial evaluating perioperative clinical outcomes and costs. Clinics 73(1):522–528. 10.6061/clinics/2017/e522s 10.6061/clinics/2017/e522s PMC6131215 30281698

[24] Sinha R, Bana R, Sanjay M (2019) Comparison of robotic and laparoscopic hysterectomy for the large uterus. J Soc Laparoendosc Surg 23(1):1–7. 10.4293/JSLS.2018.00068 10.4293/JSLS.2018.00068 PMC6328360 30675091

[25] Liu CH, Lee YC, Chien-Fu Lin J, Chan IS, Lee NR, Chang WH, Liu WM, Wang PH (2019) Radical hysterectomy after neoadjuvant chemotherapy for locally bulky-size cervical cancer: a retrospective comparative analysis between the robotic and abdominal approaches. Int J Environ Res Public Health 16(3833):2–19. 10.3390/ijerph16203833 10.3390/ijerph16203833 PMC6843229 31614465

[26] Soto E, Ha Luu T, Liu X, Magrina JF, Wasson MN, Einarsson JI, Cohen SL, Falcone T (2017) Laparoscopy vs. robotic surgery for endometriosis (LAROSE): a multicenter, randomized, controlled trial. Fertil Steril® 107:996–1002. https://www.fertstertdialog.com/users/16110 10.1016/j.fertnstert.2016.12.033 28238489

[27] Page et al (2021) The PRISMA 2020 statement: an updated guideline for reporting systematic reviews. BMJ 372:n71. 10.1136/bmj.n71 33782057 10.1136/bmj.n71 PMC8005924

[28] Braun V, Clarke V (2006) Using thematic analysis in psychology. Qual Res Psychol 3(2):77–101. 10.1191/1478088706qp063oa

[29] Gota T, Tomio K, Kurose T, Saito R, Nara R, Kin S, Hoshiba M, Ogata Y, Nakanishi M, Takamoto M, Sadatsuki M, Oishi H (2022) The current status of robotic surgery for endometrial cancer in Japan. Glob Health Med 4(1): 21–25. 10.35772/ghm.2021.01077 10.35772/ghm.2021.01077 PMC8884040 35291204

[30] Deimling TA, Eldridge JL, Riley KA, Kunselman AR, Harkins GJ (2017) Randomized controlled trial comparing operative times between standard and robot-assisted laparoscopic hysterectomy. Int J Gynaecol Obstet 136(1):64–69. 10.1002/ijgo.12001 28099699 10.1002/ijgo.12001 PMC5245181

[31] Hitesh C, Amin BA, Simona C, Inderbir S, Talha Bin E (2022) Robotics in surgery: current trends. Ann Med Surg 81:104375. 10.1016/j.amsu.2022.104375 10.1016/j.amsu.2022.104375 PMC9424352 36051814

[32] Wang J, Li X, Wu H, Zhang Y, Wang F (2020) A meta-analysis of robotic surgery in endometrial cancer: comparison with laparoscopy and laparotomy. Hindawi Dis Markers 2503753:10. 10.1155/2023/9790832 10.1155/2020/2503753 PMC7212337 32454902

[33] Bankar GR, Keoliya A (2022) Robot-assisted surgery in gynecology. Cureus 14(9):e29190. 10.7759/29190 10.7759/cureus.29190 PMC9572807 36259016

[34] Redondo-Saenz D, Cortes-Salas C, Parrales-Mora M (2023) Perioperative nursing role in robotic surgery: an integrative review. J Perianesth Nurs 38:636–641. 10.1016/j.jopan.2022.11.001 36754770 10.1016/j.jopan.2022.11.001

